# Trachoma prevalence surveys in 15 indigenous and non-indigenous evaluation units in Brazil, 2018–2023

**DOI:** 10.1093/inthealth/ihaf067

**Published:** 2025-07-01

**Authors:** Célia Landmann Szwarcwald, Maria de Fátima Costa Lopes, Paulo Roberto Borges de Souza Junior, Daniela Vaz Ferreira Gómez, Expedito José de Albuquerque Luna, Wanessa da Silva de Almeida, Giseli Nogueira Damacena, Joana da Felicidade Ribeiro Favacho, Norma Helen Medina, Luciano Chaves Franco Filho, Aiara Cogo, Sarah Boyd, Ana Bakhtiari, Cristina Jimenez, Sandra L Talero, Martha Idalí Saboyá-Díaz, Anthony W Solomon, Emma Harding-Esch

**Affiliations:** Institute of Scientific and Technological Communication and Information in Health, Oswaldo Cruz Foundation (Fiocruz), Rio de Janeiro 21040-900, Brazil; Secretariat for Health Surveillance and the Environment (SVSA/MS), Ministry of Health, Brasília 70719-040, Brazil; Institute of Scientific and Technological Communication and Information in Health, Oswaldo Cruz Foundation (Fiocruz), Rio de Janeiro 21040-900, Brazil; Secretariat for Health Surveillance and the Environment (SVSA/MS), Ministry of Health, Brasília 70719-040, Brazil; Department of Preventive Medicine, Medical School, University of São Paulo, São Paulo 05403-000, Brazil; Institute of Scientific and Technological Communication and Information in Health, Oswaldo Cruz Foundation (Fiocruz), Rio de Janeiro 21040-900, Brazil; Institute of Scientific and Technological Communication and Information in Health, Oswaldo Cruz Foundation (Fiocruz), Rio de Janeiro 21040-900, Brazil; Evandro Chagas Institute (IEC), Ministry of Health, Ananindeua, Pará 67030-000, Brazil; Sanitary Ophthalmology Center, Epidemiology Surveillance Center, São Paulo State Health Secretariat, São Paulo 01246-000, Brazil; Evandro Chagas Institute (IEC), Ministry of Health, Ananindeua, Pará 67030-000, Brazil; Secretariat for Indigenous Health (SESAI), Ministry of Health, Brasília 70719-040, Brazil; International Trachoma Initiative, Decatur, GA 30030, USA; International Trachoma Initiative, Decatur, GA 30030, USA; Neglected Tropical Diseases Department, Sightsavers, Haywards Heath RH16 3BW, UK; Communicable Diseases Prevention, Control, and Elimination Department, Pan American Health Organization, Washington, DC 20037, USA; Communicable Diseases Prevention, Control, and Elimination Department, Pan American Health Organization, Washington, DC 20037, USA; Global Neglected Tropical Diseases Programme, World Health Organization, Geneva 1211, Switzerland; London School of Hygiene & Tropical Medicine (LSHTM), London WC1E 7HT, UK

**Keywords:** Brazil, elimination, sanitation, survey, trachoma, trichiasis

## Abstract

**Background:**

To provide the groundwork for a future declaration of elimination of trachoma as a public health problem in Brazil, we conducted house-to-house surveys following WHO methodological guidance.

**Methods:**

An observational cross-sectional study was conducted in 10 non-indigenous and five indigenous evaluation units (EUs) from 2018 to 2023; data on six EUs are reported here for the first time. Two-stage cluster sampling was used: 30 clusters per EU, and 30 households per cluster. We estimated the prevalence of trachomatous inflammation—follicular (TF) in 1–9-y-olds and trachomatous trichiasis (TT) unknown to the health system in those aged ≥15 y. Data on sanitary conditions were collected in household interviews.

**Results:**

In all EUs, TF prevalence was below the elimination threshold (5%). TT prevalence was lower than the 0.2% threshold in 14 EUs. In ‘Noroeste Cearense’ mesoregion, TT prevalence was 0.22% (95% CI 0.06 to 0.44%), but statistical analysis showed a 58% likelihood of TT elimination in this EU. In three indigenous EUs, >10% of households had no sanitary facilities and high percentages of open defecation.

**Conclusions:**

It is highly likely that trachoma has been eliminated as a public health problem in all the EUs surveyed. The findings on sanitary conditions mandate public policies to overcome socioenvironmental inequalities.

## Introduction

Trachoma is a chronic inflammatory eye disease caused by the intracellular bacterium *Chlamydia trachomatis*, serotypes A, B, Ba and C. When the eye is infected by *C. trachomatis*, the bacteria develop within epithelial cells of the conjunctiva and produce an inflammatory response (hyperemia and formation of conjunctival follicles). Trachoma presents a constellation of signs, some of which have been standardized by the WHO in a grading scheme: trachomatous inflammation—follicular (TF), trachomatous inflammation—intense (TI), trachomatous scarring, trachomatous trichiasis (TT) and corneal opacity^1^; TF and TI are signs of ‘active trachoma’. Although most cases of active trachoma are mildly symptomatic, with only an ocular foreign body sensation and scanty discharge, repeated infections in childhood can lead to the formation of scars in the upper tarsal conjunctiva, which can progress to distortions in the eyelids (entropion) and in the position of the eyelashes (TT). In TT, the friction of the eyelashes against the eyeball can ulcerate the cornea, which reduces visual acuity to varying degrees and sometimes leads to blindness.^2^ Worldwide, trachoma is the main cause of blindness of infectious origin.^3^

Trachoma disproportionately affects deprived communities, where there is a lack of basic sanitation, inadequate hygiene and high household density.^4^ The disease is a public health problem in many underserved areas of Africa, Asia, Central America, South America and the Middle East. In April 2024, it was estimated that 103 million people lived in endemic areas worldwide.^5^ Elimination targets are expected to not be achieved by 2030 in several countries.^6^

WHO and its partners are targeting the global elimination of trachoma as a public health problem^7,8^ through implementation of the Surgery, Antibiotics, Facial cleanliness, and Environmental improvement (SAFE) strategy.^9^ The main components of SAFE are: surgery to correct TT; antibiotic treatment of cases or of the entire community (depending on TF prevalence) to reduce circulation of *C. trachomatis*; encouraging face washing among children; and the adoption of measures to improve the environment, basic sanitation and access to water.^9^

To declare elimination of trachoma, countries must meet three core criteria: (i) TF prevalence <5% among children aged 1–9 y in previously endemic areas; (ii) TT unknown to the health system prevalence <0.2% among individuals aged ≥15 y in previously endemic areas; and (iii) a defined strategy to identify and manage incident cases of TT.^10^ To estimate trachoma elimination parameters, WHO recommends a household-based survey in areas where trachoma is suspected to be endemic and where living conditions are precarious. Guidance to countries conducting household surveys on survey design has been published.^11^ Support provided to countries includes survey planning and design, training, data collection and storage, and analysis.^12,13^

In 2018, it was deemed necessary to conduct surveys in Brazil following WHO recommendations, to provide the groundwork for a future declaration of the elimination of trachoma as a public health problem in the country. In the first part of the ‘Prevalence Survey Series for the Validation of Elimination of Trachoma as a Public Health Problem in Brazil’, surveys were carried out in 10 evaluation units (EUs) of non-indigenous populations from 2018 to 2022. The EUs consisted of rural areas of Brazilian mesoregions, which were chosen by targeted selection based on their trachoma risk characteristics; results from the surveys in nine of these EUs have already been published.^14^ From 2021 to 2023, surveys were carried out in five areas with indigenous populations, allowing for identification of population groups that remain most vulnerable to trachoma-related factors, and reflecting our commitment to equity.

In this paper, we present the methodology and results of the trachoma prevalence surveys carried out in the 10 non-indigenous EUs and the five indigenous EUs in Brazil, and include, for comparison purposes, the results of the previously published work in this survey series.

## Materials and Methods

### Study design

An observational cross-sectional study was conducted during 2018–2023 to estimate TF prevalence among children aged 1–9 y, and TT unknown to the health system prevalence in the population aged ≥15 y, in suspected trachoma-endemic areas. The surveys followed WHO recommendations, based on Global Trachoma Mapping Project and Tropical Data (TD) protocols, with the support of TD and the Pan American Health Organization (PAHO).^12,13^

The project was developed by the Oswaldo Cruz Foundation (Fiocruz), the School of Medicine of the University of São Paulo, the State Health Department of São Paulo and the Ministry of Health (MoH), through the Secretariat for Health Surveillance and the Environment (SVSA), the Evandro Chagas Institute (IEC) and the Secretariat for Indigenous Health (SESAI).

### Non-indigenous population survey

In the first phase of the study, carried out during 2018–2019, nine mesoregions of non-indigenous populations were chosen by targeted selection to constitute the EUs. These mesoregions are in the North and Northeast regions of the country, have rural populations of 100 000–250 000 inhabitants, at least one endemic municipality and precarious socioeconomic and sanitation conditions. The results of surveys conducted in these EUs have previously been published.^14^

Subsequently, it was considered relevant to carry out a survey in an endemic area of the state of São Paulo, given that, historically, this state has been an important focus of trachoma in Brazil.^15^ In 2022, the ‘Litoral Sul Paulista’ mesoregion was surveyed as the 10th non-indigenous EU.

### Indigenous population surveys

In 2024, the population living in indigenous villages in Brazil was estimated at 806 300 inhabitants, corresponding to 0.37% of the Brazilian population.^16^ Healthcare for indigenous populations in Brazil is a federal government responsibility through the SESAI, which organizes its actions through the Special Indigenous Health Districts (*Distritos Sanitários Especiais Indígenas*, DSEI).

Conducting research in indigenous areas requires approval from the National Research Ethics Commission (CONEP), authorization from the National Indian Foundation (FUNAI) for research team members to enter indigenous lands and approval from the District Indigenous Health Councils (CONDISI) of the selected DSEIs to be surveyed.

The indigenous population was encouraged to participate in the research through discussion with the local CONDISI representative and community residents, with the help of indigenous health agents, sometimes through interpreters. It was explained that the research would lead to actions to eliminate trachoma as a public health problem and therefore help to prevent blindness.

**Figure 1.**
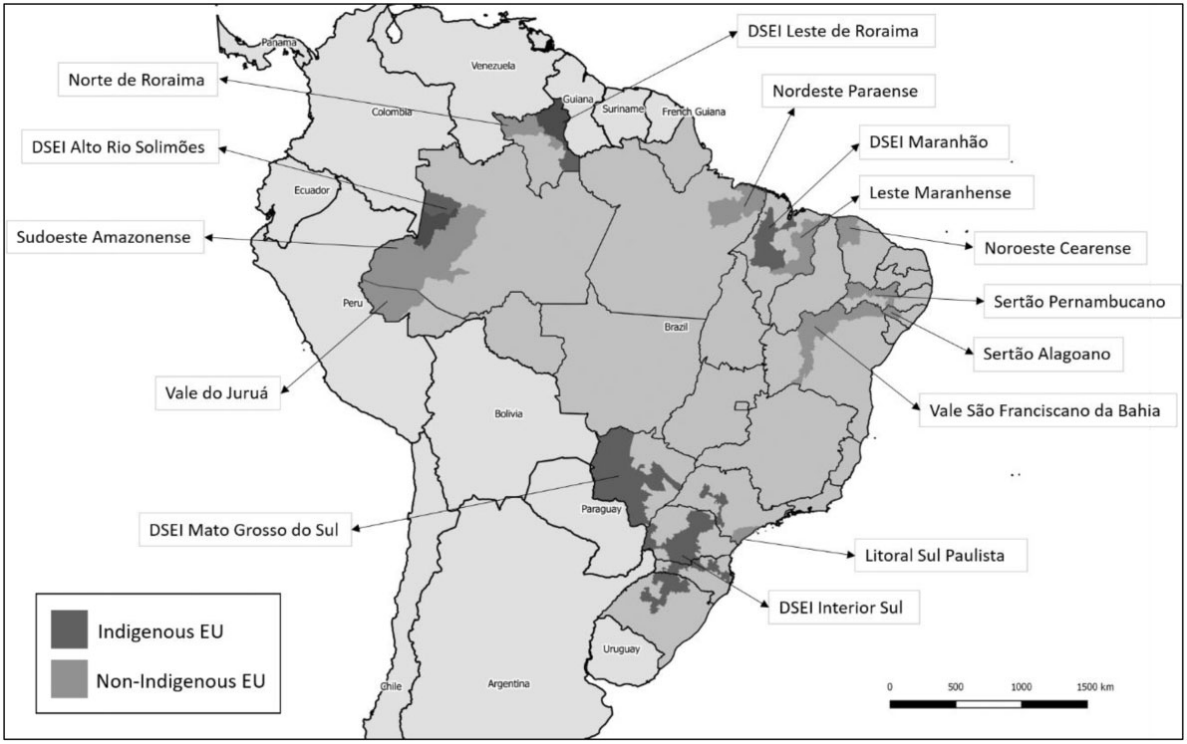
Indigenous and non-indigenous evaluation units (EUs) included in population-based trachoma prevalence surveys, Brazil, 2018–2023*. *The boundaries and names shown and the designations used on this map do not imply the expression of any opinion whatsoever on the part of the authors, or the institutions with which they are affiliated, concerning the legal status of any country, territory, city or area or of its authorities, or concerning the delimitation of its frontiers or boundaries.

Following WHO recommendations, and considering the population size, geographic location and previous data on the occurrence of trachoma, five DSEIs were chosen, by targeted selection, to be surveyed. All considered EUs are presented in the map of Brazil (Figure 1).

### Sampling plan

Calculation of the minimum sample size in all EUs followed WHO recommendations.^10,11,17^

For indigenous and non-indigenous EUs, the sample was selected with two-stage cluster sampling. In each EU, 30 clusters (primary sampling units) were selected with probability proportional to size of the resident population. In the non-indigenous population, the clusters were rural communities of the EU, while in the indigenous population, the clusters were indigenous villages of the DSEI. In the second stage, each cluster selected in the first stage was mapped and subdivided into segments of 30 neighboring homes. Using the compact segment sampling method, one to three segments were randomly selected in each cluster (community) to obtain 30 segments with 30 households in each EU, providing a total sample of 900 households in each EU.

### Case detection

Both eyes of each survey participant were examined by a trained examiner to detect clinical signs compatible with trachoma.^1^ Follicle size guide stickers were used to help with the diagnosis of TF.^18^

### Data collection and storage

These surveys were supported by TD, which assists trachoma programs to undertake trachoma surveys.^13^ A questionnaire for collecting information using smartphones and a data storage platform are part of the support offered by TD. The geographic coordinates of all the households surveyed were captured by the smartphones´ GPS, enabling the monitoring of fieldwork by both TD staff and the national team responsible for the survey.

The TD questionnaire was adapted to be applied in the Brazilian surveys. Questions regarding the environmental sanitation and hygiene conditions were asked of a key informant in each household. Data were collected regarding the source of drinking water and water for hygiene purposes and the destination of waste and solid residues. At an individual level, the questionnaire included a section dedicated to demographic information about household residents, as well as the results of clinical exams.

All residents aged ≥1 y in the households selected for inclusion were examined for trachoma. The teams recorded details of absentees, and there was at least one return visit to try to examine them. Both the interview records and the results of the external ocular examination of both eyes were stored on a mobile phone and uploaded to the TD data storage platform. Where cases of TT were detected, additional questions were asked to investigate whether the health service was aware of the cases to establish if the TT was ‘known to the health system’.

### Fieldwork

Fieldwork teams were composed of an examiner trained in the clinical diagnosis of trachoma in accordance with WHO and TD standards,^1,19^ an interviewer responsible for recording the questionnaire responses and a local collaborator who accompanied the team to facilitate the study in the selected areas. The teams were supervised by Fiocruz and TD staff, who monitored the progress and quality of the fieldwork. All team members received prior training on the general activities of the survey, objectives, design and procedures. The result of the external ocular examination was communicated to the individual at the time it was performed, as well as to the parents or guardians of examined children.

All cases of TF and TI identified in fieldwork were treated with azithromycin, in accordance with MoH guidelines. All TT cases identified in the study were reported to the state and municipal health departments, or in the case of indigenous individuals to SESAI and the DSEI, who were responsible for referring them to ophthalmology services for further evaluation and management.

### Data analysis

In each EU, prevalence estimates of TF and TT and their respective 95% CIs were estimated, considering the effects of the sampling plan and the EU population distribution by age group (for TF) and by age group and gender (for TT).^13^

The household interview data were analyzed in accordance with the WHO/UNICEF Joint Monitoring Program for Water Supply, Sanitation and Hygiene, with some additional healthcare and education analyses.

### Ethical aspects

The project was approved by the National Research Ethics Committee (CONEP) of the National Health Council, Brazilian Ministry of Health (logged under protocol no. 2742820), and by the Ethics Committee of PAHO (2018–06–0045). TD support for the surveys was approved by the London School of Hygiene & Tropical Medicine Ethics Committee (reference 16105).

## Results

In each EU, approximately 900 households were surveyed. In all 15 EUs, 58 195 individuals aged ≥1 y were registered as living in the selected households, of whom 51 526 (88.5%) were examined for trachoma.

As shown in Table 1, TF prevalence in all 15 EUs was below the WHO 5% elimination threshold. In the non-indigenous EUs, TF prevalence was quite close to zero, ranging from 0 to 1%. In the indigenous EUs, TF prevalence ranged from 0 to 3.3%.

**Table 1.** Prevalence of trachomatous inflammation—follicular (TF) in children aged 1–9 y, by evaluation unit (EU), Brazil, 2018–2023

EU	Number of children aged 1–9 y examined	Number of TF cases	Adjusted TF prevalence (%)*	95% CI
Non-indigenous EUs				
Vale do Juruá^1^	682	0	0.0	0.0–0.0
Sudoeste Amazonense^1^	1045	8	0.7	0.1–1.5
Norte de Roraima^1^	591	5	0.6	0.1–1.4
Nordeste Paraense^1^	764	9	1.0	0.3–1.9
Leste Maranhense^1^	620	1	0.1	0.0–0.4
Noroeste Cearense^1^	554	3	0.6	0.0–1.5
Sertão Pernambucano^1^	466	2	0.2	0.0–0.5
Sertão Alagoano^1^	648	0	0.0	0.0–0.0
Vale São Franciscano da Bahia^1^	614	1	0.1	0.0–0.2
Litoral Sul Paulista	357	1	0.2	0.0–0.6
Indigenous EUs				
DSEI Interior Sul	685	0	0.0	0.0–0.0
DSEI Mato Grosso do Sul	682	0	0.0	0.0–0.0
DSEI Alto Rio Solimões	1335	41	2.6	1.2–4.1
DSEI Maranhão	1275	46	2.9	1.4–5.0
DSEI Leste de Roraima	1131	42	3.3	1.1–5.0

DSEI: Distrito Sanitário Especial Indígena (Special Indigenous Health District).

* Calculated by Tropical Data and adjusted for age, considering the population of the 2010 Brazilian Demographic Census as the standard.

^1^Published in Szwarcwald et al.^14^

The comparison of TF prevalence in children aged 1–9 y living in the non-indigenous ‘Sudoeste Amazonense’ mesoregion (0.7%) and the indigenous DSEI ‘Alto Rio Solimões’ (2.6%), both located in the same municipalities of the state of Amazonas, revealed a fourfold higher TF prevalence in the indigenous EU (Table 1). Likewise, the TF prevalence in the indigenous district ‘Maranhão’ was 2.9%, higher than that found in the non-indigenous EU ‘Leste Maranhense’ (0.1%), both EUs in the state of Maranhão.

Prevalence of TT unknown to the health system and the respective 95% CIs are presented in Table 2. In all 10 non-indigenous EUs, TT prevalence was below the WHO 0.2% threshold, except in the ‘Noroeste Cearense’, which showed a TT prevalence of 0.22% (95% CI 0.06 to 0.44%). In the five indigenous EUs, TT prevalence ranged from 0 to 0.12%.

**Table 2.** Prevalence of trachomatous trichiasis (TT) in individuals aged ≥15 y, by evaluation unit (EU), Brazil, 2018–2023

EU	Number of individuals aged ≥15 y examined	Number of cases of TT unknown to the health system*	Adjusted prevalence of TT unknown to the health system (%)**	95% CI
Non-indigenous EUs				
Vale do Juruá^1^	2002	0	0.00	0.00–0.00
Sudoeste Amazonense^1^	2226	0	0.00	0.00–0.00
Norte de Roraima^1^	1663	1	0.05	0.00–0.15
Nordeste Paraense^1^	2312	0	0.00	0.00–0.00
Leste Maranhense^1^	2035	0	0.00	0.00–0.00
Noroeste Cearense^1^	1971	6	0.22^a^	0.06–0.44
Sertão Pernambucano^1^	2058	2	0.05	0.00–0.12
Sertão Alagoano^1^	2230	0	0.00	0.00–0.00
Vale São Franciscano da Bahia^1^	1997	2	0.05	0.00–0.13
Litoral Sul Paulista	1683	0	0.00	0.00–0.00
Indigenous EUs				
DSEI Interior Sul	2016	0	0.00	0.00–0.00
DSEI Mato Grosso do Sul	1804	0	0.00	0.00–0.00
DSEI Alto Rio Solimões	2532	1	0.01	0.00–0.02
DSEI Maranhão	2347	7	0.12	0.02–0.24
DSEI Leste de Roraima	1985	1	0.03	0.00–0.09

* Unknown to the health system excludes cases where management has been previously offered by a health professional.

** Calculated by Tropical Data and adjusted for gender and age, considering the population of the 2010 Brazilian Demographic Census as the standard.

^1^Published in Szwarcwald et al.^14^

a The standard statistical approach showed a 58% likelihood of TT prevalence being below the elimination threshold (<0.2%).

The indicators related to sanitary conditions are presented in Table 3. In the non-indigenous population, the proportion of households with no sanitary facilities was >10% in five of the 10 EUs, while in the indigenous population, values >10% were found in three of the five EUs. The highest percentages of households without sanitation facilities occurred in the two EUs located in the state of Maranhão, ‘Leste Maranhense’ (52%) and the DSEI ‘Maranhão’ (65%).

**Table 3.** Indicators related to sanitary conditions and access to water, by evaluation unit (EU), Brazil, 2018–2023

EU	No sanitary structure (%)	Open disposal of feces (%)	Availability of drinking water (%)	Availability of water to face washing (%)	Availability of soap near the toilet (%)
Non-indigenous EUs					
Vale do Juruá	29.0	8.9	63.0	65.0	30.1
Sudoeste Amazonense	8.9	2.4	54.9	51.1	16.6
Norte de Roraima	7.3	0.8	69.2	74.2	56.3
Nordeste Paraense	6.2	5.3	49.3	66.1	38.4
Leste Maranhense	52.1	6.8	50.4	58.2	20.6
Noroeste Cearense	7.0	0.7	81.4	91.0	74.2
Sertão Pernambucano	15.9	2.8	66.6	58.9	66.1
Sertão Alagoano	20.1	2.9	50.7	44.4	64.1
Vale São Franciscano da Bahia	11.9	4.6	85.7	93.6	69.7
Litoral Sul Paulista	1.4	0.6	98.3	99.8	82.0
Indigenous EUs					
DSEI Interior Sul	18.6	4.4	88.7	89.6	54.4
DSEI Mato Grosso do Sul	5.7	13.4	70.4	70.6	58.0
DSEI Alto Rio Solimões	5.9	8.9	93.8	90.8	45.3
DSEI Maranhão	64.7	27.3	71.2	67.2	12.1
DSEI Leste de Roraima	10.3	11.4	91.6	94.0	60.3

High percentages of open disposal of feces were identified in some EUs. In non-indigenous EUs, the percentages were <9%. In indigenous EUs, there was a greater range of variation, from 4 to 27% (Table 3). The proportions of households with available drinking water varied from 49 to 98% among non-indigenous EUs, and from 70 to 94% among indigenous EUs. Regarding the availability of water for face washing, the percentages varied from 44 to nearly 100% in non-indigenous EUs, and from 67 to 94% in indigenous EUs. With regards to the availability of soap near the toilet, ‘DSEI Maranhão’ had the lowest percentage (12%) and ‘Litoral Sul Paulista’ had the highest (82%).

Indicators of healthcare, children's education and household density are presented in Table 4. Proportions of children aged 5–14 y attending school were >94% in all 15 EUs. The average number of residents per household was 3.7 in the 10 non-indigenous EUs, and 4.6 in the five DSEIs. The percentage of households that reported having received monthly visits from community health agents had a median of 67%. Regarding monthly visits by indigenous sanitation agents, the percentages were low in all indigenous EUs, ranging from 14 to 50%.

**Table 4.** Indicators related to education, healthcare and household density, by evaluation unit (EU), Brazil, 2018–2023

EU	Children aged 5–14 y attending school (%)	Average number of residents per household	Monthly visits from community health agents (%)	Monthly visits from indigenous sanitation agents (%)
Non-indigenous EUs				
Vale do Juruá	99.4	4.0	73.1	-
Sudoeste Amazonense	97.8	4.8	77.6	-
Norte de Roraima	94.8	3.3	47.6	-
Nordeste Paraense	99.8	4.4	65.1	-
Leste Maranhense	99.5	3.8	77.2	-
Noroeste Cearense	98.8	3.4	67.0	-
Sertão Pernambucano	99.4	2.9	87.5	-
Sertão Alagoano	98.2	3.5	70.4	-
Vale São Franciscano da Bahia	99.2	3.6	71.6	-
Litoral Sul Paulista	97.6	2.8	31.4	-
Indigenous EUs				
DSEI Interior Sul	96.9	3.7	62.3	16.6
DSEI Mato Grosso do Sul	95.5	3.7	55.8	14.0
DSEI Alto Rio Solimões	97.6	5.8	63.8	21.2
DSEI Maranhão	96.8	5.0	88.6	50.7
DSEI Leste de Roraima	99.0	4.6	43.6	24.9

## Discussion

This study augments the data published previously by Szwarcwald et al.,^14^ which presented results from the nine non-indigenous population EUs surveyed during 2018–2019. The results from those surveys are also included in the current paper to provide a complete account of the non-indigenous EUs, because we conducted a survey in one additional non-indigenous EU (‘Litoral Paulista’) after the COVID-19 pandemic. Having all relevant recent data compiled here should aid comparisons of prevalence and variance estimates between non-indigenous and indigenous EUs and may serve as a reference for other countries in the region.

In the first stage of mapping the epidemiological situation of trachoma in Brazil, the target of eliminating trachoma as a public health problem was achieved in all nine EUs surveyed, except in the ‘Noroeste Cearense’ mesoregion, for which a TT prevalence of 0.22% was estimated. However, traditional statistical inference showed that the probability of TT being below the elimination threshold (<0.2%) was 58%, indicating a high likelihood of trachoma elimination in this EU. After completing the research, surgical treatment was offered to all TT cases. However, none of the TT cases detected in the survey agreed to undergo surgery. The patients were older people and did not have a family member to accompany them during the surgical process.

Model-based geostatistics (MBG) was also used to assess the elimination of trachoma as a public health problem in Brazil and other countries.^20^ In addition to age and gender, the effects of spatial correlation were taken into account. In the MBG approach, six EUs of Brazil's North and Northeast were included. TF prevalence met the elimination target across these EUs. For TT, geostatistical analysis showed that the probability of TT being below the elimination threshold was >90% in three out of six EUs, for two of them the probabilities were 85 and 78%, and for ‘Noroeste Cearense’ the probability was 70%, suggesting a high likelihood of trachoma elimination across these EUs. In other words, the geostatistical analysis confirmed the general impression for TF and TT obtained by the traditional approach and additionally suggested that TT was likely below the threshold in ‘Noroeste Cearense’.

In 2022, the inclusion of the EU ‘Litoral Sul Paulista’ affirmed that trachoma has probably been eliminated as a public health problem in the non-indigenous Brazilian population; this conclusion is based on our belief that the areas overwhelmingly most likely to have trachoma were surveyed, and its absence from those populations can be extrapolated to absence from the non-indigenous population throughout the country.

Among the likely explanations for the clear improvements compared with historical data in the trachoma situation of Brazil's non-indigenous population^21^ is the strengthening of trachoma surveillance actions, the implementation of the Family Health Strategy in the late 1990s,^22^ which resulted in the expansion of primary healthcare and in the use of health services, and the income distribution programs, which have reduced socioeconomic disparities in morbidity and mortality in Brazil, and been associated with reductions in endemic diseases.^23^ Additional contributions came from programs aimed at promoting access to water for human consumption, such as the program of cisterns (i.e. containers to receive and conserve rainwater), in which priority was given to low-income water-insecure rural families.^24^

**Figure 2.**
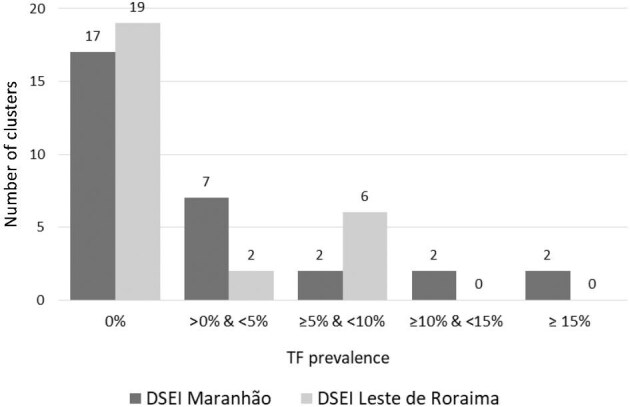
Prevalence of trachomatous inflammation—follicular (TF) by cluster, in the evaluation units ‘DSEI Maranhão’ and ‘DSEI Leste de Roraima’. DSEI: Distrito Sanitário Especial Indígena (Special Indigenous Health District).

In 2021, the second stage of trachoma mapping in Brazil began in five indigenous EUs. Data from previous trachoma prevalence surveys carried out in some DSEIs indicated prevalence ranges higher than those of Brazil's non-indigenous populations.^25,26^ In the current survey series, TF prevalence was below the elimination threshold in all indigenous EUs surveyed. Nevertheless, in two indigenous EUs (‘DSEI Leste de Roraima’ and ‘DSEI Maranhão’), TF prevalence showed greater relative heterogeneity among villages surveyed, with wide CIs in the overall estimate and a concentration of TF and TT cases in some specific indigenous communities. In these communities, postelimination surveillance initiatives will be implemented, including expanded surveillance for TF and TT, treatment to children with TF and their household contacts, as well as surgical management for individuals with TT. Although there was heterogeneity in TF prevalence within these two EUs, analysis of cluster-level prevalence shows an exponential frequency distribution, as exemplified for DSEI Maranhão and DSEI Leste de Roraima (Figure 2). This is consistent with trachoma being on its way to disappearing from these two EUs.^27^

Regarding TT unknown to the health system, prevalence estimates were <0.2% in all indigenous EUs. However, ‘DSEI Maranhão’ presented a total of seven TT cases for which management had not previously been offered by a health professional, and six of the seven cases were located precisely in the same clusters where the highest TF prevalence estimates were found among children aged 1–9 y. One interpretation of this result is that the trachoma situation in these indigenous communities has persisted over the years, and the current TT cases are most likely in individuals who had repeated infections in childhood years ago. Thus, although public policies focused on reducing social disparities and strengthening trachoma surveillance actions have contributed to the reduction of trachoma in Brazil, some indigenous communities still face persistent trachoma problems and extreme socioenvironmental vulnerability, evidencing the importance of sustained postelimination surveillance strategies, particularly in these areas.

Additionally, in four of the five indigenous EUs surveyed, <25% of households received a monthly visit from the indigenous sanitation agent. Thus, there is a clear gap between strategy and practice in periodic visits by indigenous health agents, indicating failures in the processes by which communities and their inhabitants build knowledge and attitudes aimed at acquiring good sanitation conditions and hygiene. Following the examples of countries that have recently been validated as having eliminated trachoma as a public health problem,^28^ it is necessary to implement comprehensive, inclusive and intersectoral programs that leave no one behind. These public policies will certainly bring long-term benefits for a healthier Brazil and for accelerating the achievement of the Sustainable Development Goals.^29^

Regarding education, we were encouraged by the percentage of children aged 5–14 y who were reportedly attending school, which was ≥95% in all 15 EUs surveyed. These children are expected to experience the impact of education in acquiring healthier lifestyle behaviors, potentially preventing trachoma in the future.^30^

Among the limitations of our work is the lack of precision in TT prevalence estimates at EU level, because the sample size is calculated to estimate TF prevalence,^13^ but is not sufficiently large to precisely estimate TT prevalence, generally close to zero. Another caveat is that some DSEIs were excluded from the study, as they had carried out antibiotic mass drug administration 3 y prior to the survey. In the future, surveys should be conducted in these DSEIs to estimate trachoma prevalence and undertake other intersectoral initiatives in parallel.

### Conclusions

These data from surveys conducted in 15 EUs, using standard statistical approaches, show that it is highly likely the goals of eliminating trachoma as a public health problem have been achieved in all EUs surveyed. Additionally, the findings regarding sanitary conditions and hygiene habits provided important support for the implementation of health education strategies and public policies to overcome environmental and social inequalities. Intercultural dialogue practices could be adopted in the educational and participatory construction of knowledge for trachoma prevention and health promotion.

## Data Availability

The data underlying this article were provided by Brazilian Ministry of Health under licence / by permission. Data will be shared on request to the corresponding author with permission of Brazilian Ministry of Health.
